# Psychological Resilience among Orang Asli Youths in Selangor during COVID-19 and Its Associated Factors

**DOI:** 10.21315/mjms2024.31.3.16

**Published:** 2024-06-27

**Authors:** Nurjuliana Noordin, Nik Nairan Abdullah, Raudah Mohd Yunus

**Affiliations:** Department of Public Health Medicine, Faculty of Medicine, Universiti Teknologi MARA, Selangor, Malaysia

**Keywords:** Orang Asli, COVID-19, resilience, indigenous people, pandemic

## Abstract

**Background:**

Coronavirus disease (COVID-19) has affected everyone and stress-related mental health issues affect young people more than other groups, including marginalised populations. As a result of this pandemic, society is being urged to examine indigenous psychological resilience, especially among Orang Asli (OA) communities in Malaysia. Hence, this study aims to identify factors associated with psychological resilience among OA youths of Kampung Orang Asli (KOA) in Gombak during the COVID-19 pandemic.

**Method:**

A cross-sectional study comprising OA communities was conducted between January 2022 and September 2022 in the Gombak District, Selangor. A self-administered online questionnaire using Google Forms and a self-administered printed questionnaire was used to collect data from youths aged 18 years old–24 years old. The Malay version of Conner-Davidson Resilience Scale-25 (CD-RISC-25) was used to assess psychological resilience. Data analysis was performed using SPSS version 28.0, and multiple linear regression analyses were conducted to evaluate the associated factors and their significance level.

**Result:**

A total of 158 participants were involved in this study. The mean score for psychological resilience was estimated at 69.28 (SD = 14.52). The social relationships domain recorded the highest mean score for quality of life (71.54, SD = 13.72). Meanwhile, the total mean score for self-esteem fell into the high-level category (35.77, SD = 4.94), and the domain of relationship and family dynamics under family environment scored the highest mean score of 18.83 (SD = 2.89). Psychological resilience was associated with youths of KOA Batu 12 (*β* = −14.274, *P* < 0.05), KOA Ulu Batu (*β* = −17.789, *P* < 0.05), less than four siblings (*β* = −6.495, *P* < 0.05), owner of residential property (*β* = −7.543, *P* < 0.05), high self-esteem (*β* = 0.612, *P* < 0.05) and good relationship and family dynamic (*β* = 1.391, *P* < 0.05).

**Conclusion:**

Developing interventions aimed at psychological resilience determinants may assist OA youths in coping with future threats.

## Introduction

World health, economic, environmental and social sectors were devastated by the coronavirus disease 2019 (COVID-19) outbreak (1), causing unprecedented suffering, impacting the economy and claiming billions of lives (2). The COVID-19 pandemic negatively affected all demographic groups, but the poor, elderly, disabled, young and indigenous groups were most negatively affected (3). Individuals should offer psychological support to one another and work on developing coping techniques to lessen the pandemic’s impact (4). To improve medical care and treatment, it is important to measure individual mental health outcomes and protective variables, such as psychological resilience (4).

The American Psychological Association (APA) defines resilience as the ability to cope well with adversity, trauma, tragedy, threats or severe sources of stress, such as family and relationship issues, substantial health concerns, workplace stressors and financial stressors (5). Complicated biological occurrences like pandemics can affect social, economic, political and psychological aspects (6). A comparison of the psychological resilience score between white Americans (77.4) and non-white Americans (76.7) over the first few weeks of COVID-19 found that the mean psychological resilience score for white Americans was 66.84, compared with 76.7 for non-white Americans (7). This decrease negatively impacted the Americans’ perceptions of their psychological resilience, possibly due to the abrupt shifts in their emotional perception (7).

During catastrophes, indigenous populations such as Malaysia’s Orang Asli (OA) find resilience using centuries-old catastrophe responses (8, 9). To defend themselves and reduce the impact of COVID-19, they have sought guidance from nature and social collaboration for a long time (10, 11). However, individuals with lower stress and adaptability thresholds became susceptible to the pandemic, whereby tribal opposition and displacement jeopardised OA’s way of life (12, 13).

Peninsular Malaysia’s indigenous OA group has a unique history, religion, socioeconomic status and beliefs that set them apart from other ethnic groups (14). They are a diverse group of clans and tribes with distinct languages, cultures and customary lands. Eighteen OA subgroups fall within the Negrito, Senoi and Proto-Malay groups (15), comprising approximately 0.7% of Peninsular Malaysia’s population (31,950,000) (15, 16). However, they are unified because they are distinct from Peninsular Malaysia’s three largest ethnic groups (Malay, Chinese and Indian) and have historically been marginalised in social, economic and cultural issues (17). OA’s main challenge today is being displaced from their ancestral lands (16).

Many OAs were forced into poverty during the COVID-19 pandemic lockdown because they were unable to work or sell their agricultural products (13). A lack of demand for harvested crops during the pandemic also delayed the sale of harvested crops, delaying the provision of financial resources and government support. (18). Furthermore, health concerns, lack of water and sanitation, and proximity, particularly in government resettlements, make OA more vulnerable to COVID-19 community spread (19). This is demonstrated by the fact that August 2021 had the highest transmission month, with 2,389 cases and 107 deaths (20). This was deemed important since, while accounting for 0.5% of Malaysia’s total population, OA was responsible for 0.2% of new cases (*n* = 1,422,005) and nearly 0.7% of mortality statistics (*n* = 16,087) (20).

Pandemics challenge the world, particularly in understanding and evaluating the psychological resilience of indigenous populations, particularly OA in Malaysia (21). Youths are more susceptible to the mental health effects of stress due to physiological and behavioural changes (21). Due to the lack of resilience research in this age group, there is a large gap between the effects among marginalised youth and how resilience can be enhanced in this community (22). Indigenous knowledge and practices should be studied to assist this population in making the best use of their assets to become more resilient. This study aims to determine the psychological resilience level of OA youth in Gombak during the COVID-19 pandemic and identify factors associated with this resilience.

## Methods

A cross-sectional study involved eight OA villages in the Gombak District, Selangor, Malaysia between January 2022 and September 2022. The inclusion criteria included OA youths aged between 18 years old and 24 years old from Kampung Orang Asli (KOA) Batu 12, KOA Batu 16, KOA Ulu Batu, KOA Sungai Buloh, KOA Kuang, KOA Bukit Lagong, KOA Sungai Relang and KOA Kemensah in the Gombak District, Selangor, and those who read, write and speak in Malay. Individuals with mental illness or psychiatric disorder history were excluded from the study. The Gombak District was selected for the study as it was a suburb with good internet access and had an indigenous OA settlement with an OA Museum (23).

A universal sampling strategy was applied as it was still the pandemic period and because of the poor responses from the population data collection. Information needed to estimate the sample size was obtained from the existing literature; the standard deviation of the resilience score was 13.0 (24). Using the Epitools software, the sample size required was 163, with a confidence level of 0.95, a standard deviation (SD) of 13.0 and a desired precision of 2 (25). By adjusting a 20% non-response rate, the minimum sample required for this study was 196.

## Data Collection Method

Several strategies were implemented to promote response and participation in light of the crisis. In the first method, a consent form was attached to an online self-administered questionnaire through Google. The questionnaire was distributed to all village participants with available WhatsApp contact numbers. In addition to the self-administered online questionnaires, village representatives were sent hard copies (in envelopes) and were instructed: i) to distribute the questionnaire to youths aged between 18 years old and 24 years old; ii) to collect the completed questionnaire and put them all into the envelopes provided; and iii) to return the questionnaires in the envelope back to the researcher. The study maintained strict confidentiality and data anonymity.

As independent variables, this study investigated the demographic details, quality of life (QOL), self-esteem, mental health and family environment. The questionnaire was adopted and prepared in Malay. Six main instruments were used for data collection under six main sections: A) socio-demographic background, B) resilience, C) QOL, D) self-esteem, E) mental health and F) family environment. The questionnaire was pre-tested with 30 youths to ascertain its clarity, order of questions and consistency.

In Section A (socio-demographic), participants were asked about their age, gender, sub-ethnic group, village name, education level, marital status, household income, employment status, family structure, family size, religiosity, housing tenure and medical history.

Section C (QOL) is the Malaysian version of the WHOQOL-BREF questionnaire consisting of 26 questions to assess the QOL (26). Of 26 questions, 2 were evaluated for QOL and overall health satisfaction. The remaining 24 questions or items were divided into four domains: i) physical, ii) psychological, iii) social and iv) environmental. Based on a 5-point Likert scale, the responses for each question were classified and scored from 1 to 5. The score of each question for each domain was obtained as a summarised domain score, and finally, all the scores were linearly transformed according to the WHOQOL-BREF guidelines (27). High domain scores indicate higher QOL levels than low domain scores. The Cronbach alpha (24 items) for questions 3–26 was estimated to be 0.89.

To assess the level of self-esteem in section D, participants were asked to complete the validated Malay version of the Rosenberg Self-Esteem (RSE) scale to evaluate the self-esteem levels (28). The scale consisted of 10 items on a 5-point Likert scale (strongly agree, agree, no idea, disagree and strongly disagree) with an equal number of positive (1, 3, 4, 7 and 10) and negative (2, 5, 6, 8 and 9) items. The Cronbach’s alpha was 0.8. The negative scoring items indicated negative self-esteem, while the positive ones indicated positive self-esteem. The scoring method is calculated by summing up all the items in the scale; a higher score indicates an individual higher level of self-esteem.

The validated Malay version of the Depression, Anxiety, Stress Scale 21 (DASS21) was applied as the study tool to evaluate the mental health levels of respondents’ depression, anxiety and stress in section E. The reliability (internal consistency) with the overall Cronbach alpha values was robust at 0.90, with depression, anxiety and stress scale values of 0.84, 0.74 and 0.79, respectively (29). This section comprised of 21 questions. Respondents were asked to rate their interactions on a 4-point Likert scale ranging from 0 (Did not apply to me at all) to 3 (Applied to me much of the time). The cut-off score for each of the DASS21 variables is as follows: Depression: normal (0–4), mild (5–6), moderate (7–10), severe (11–13) and extra severe (14+); Anxiety: normal (0–3), mild (4–5), moderate (6–7), severe (8–9) and extra severe (10+); Stress: normal (0–7), mild (8–9), moderate (10–12), severe (13–16) and extra severe (17+).

On the other hand, section F, which represents the family environment, utilised the bahasa Malaysia version of the Family Environmental Scale (FES) to measure Malaysian adolescents’ perception of their family environment (30). Internal accuracy for this analysis ranged from 0.10 to 0.70 on the Cronbach’s alpha scale. It contained 30 questions with a 4-point Likert scale ranging from highly agree = 4, agree = 3, disagree = 2 to highly disagree = 1.

Meanwhile, section B utilised the Malay version of Conner-Davidson Resilience Scale-25 (CD-RISC-25) to measure psychological resilience, the study’s dependent variable (31). CD-RISC-25 is a self-report measure consisting of 25 items on a 5-point Likert scale: 0 = not true at all, 1 = rarely true, 2 = sometimes true, 3 = often true and 4 = true nearly all of the time. This scale assesses five resilience factors: i) personal competence, high standards and tenacity; ii) trust in one’s instincts, tolerance of negative affect and strengthening effects of stress; iii) positive acceptance of change and secure relationships; iv) control and iv) spiritual influences. The total score for all 25 items ranges from 0 = indicating a lesser degree of resilience to 100 = indicating a larger degree of resilience. A satisfactory overall internal consistency was established using the general population sample with an alpha coefficient of 0.89 (31).

This analysis included an evaluation of mental well-being using the DASS-21. Therefore, participants with mild to highly severe DASS-21 scores were contacted and offered a referral for further evaluation and investigation at the nearest health clinic. Contact details of the Befrienders (Kuala Lumpur), the Malaysian Association and Adolescent Health (MAAH), and Cafe@TEEN under the Ministry of Women, Family and Community Development were stated on both questionnaires (online and hard copy) offering them counselling and help for mental health.

## Data Analysis

Data analysis was performed using the Statistical Package for the Social Sciences (SPSS) version 28.0. The characteristics of the study population, psychological resilience level, QOL, self-esteem, depression, anxiety and stress score, and family environment (frequency distribution, central tendencies, measure of distribution) were assessed using descriptive statistics. To examine psychological resilience factors, researchers used multiple linear regression (enter method) analyses to assess the associations and measure the level of statistical significant. Any *P* < 0.05 was considered statistically significant.

## Result

### Sociodemographic

The study involved 240 youths from KOA Batu 12, KOA Batu 16, KOA Ulu Batu, KOA Sungai Buloh, KOA Kuang, KOA Bukit Lagong, KOA Sungai Relang and KOA Kemensah in the Gombak District of Selangor, Malaysia. Of these, only 163 youths responded to the questionnaire, indicating a total response rate of 81.9%. However, only 158 study participants remained after examining and cleaning the data; five had to be removed since they were duplicates. As the percentage of missing data was less than 5%, the imputation method using mean and mode substitution was used to fill in the missing values. The village of Kampung Orang Asli (KOA) Batu 16, KOA Sungai Relang and KOA Kemensah were grouped since they have a relatively low population, accounting for 5.1% (*n* = 8) of the total participants. Table 1 summarises the baseline characteristics of the study participants.

The mean age of participants was 20.96 years old with a SD of 1.96 years old. Most participants were females (65.8%) and ethnicity differed in being Proto-Malay (72.2%), Temuan (69.0%), with a majority from KOA Batu 12 (43%), completed secondary education (63.3%) and single (72.2%). Almost all (97.5%) participants hailed from families with an annual income of less than RM4,500. The majority of them are unemployed (53.2%), live with both of their parents (78.5%), have four or more siblings (77.2%) and are Islamic (55.1%), whereas 84.2% were homeowners.

### Psychological Resilience, QOL, DASS-21, Self-Esteem and Family Environment

Table 2 summarises the findings for the psychological resilience, QOL, self-esteem, DASS-21 and family dynamic scores. The low and high psychological resilience groups were applied to categorise the resilience levels (31). The median score for psychological resilience was 72.50, with the first (Q1), second (Q2), third (Q3) and fourth (Q4) quartiles scores ranging from 0–59, 60–73, 73–77 and 78–100, respectively (31). In our study only the Q4 was regarded as a high resilience group, while the Q1, Q2 and Q3 were considered low resilience groups (31). The mean score for psychological resilience in this study was estimated at 69.28 (SD = 14.52), indicating overall low psychological resilience among the participants. Participants reported good QOL and general health satisfaction, with mean scores of 3.95 (SD = 0.76) and 4.15 (SD = 0.69), respectively. The domain of social relationships measured the highest mean QOL score of 71.54 (SD = 13.72), followed by the environmental with mean score of 68.39 (SD = 13.29), psychological with mean score of 60.76 (SD = 16.83) and physical health with mean score of 59.10 (SD = 10.43) determinants. The total mean score for self-esteem was 35.77 (SD = 4.94). A cut-off point of 25 was used to categorise levels of self-esteem into low (10–25) and high (25–40) levels, with the mean score of participants falling into the high-level category (33). According to the psychological well-being profile, 35 (22.2%) of the participants exhibited mild to extremely severe symptoms of depression, 37 (23.4%) suffered mild to extremely severe symptoms of anxiety and 21 (13.3%) indicated mild to extremely severe symptoms of stress. Relationship and family dynamics recorded the highest mean score among family environments, at 18.83 (SD = 2.89), followed by togetherness and harmony at 18.13 (SD = 3.10), religion and traditional practices at 17.27 (SD = 3.18) and conflict at 16.55 (SD = 3.84). The expression had the lowest mean score among family environments, with only 16.01 (SD = 3.75).

### Factors Associated with Psychological Resilience

Table 3 summarises the results of the linear regression model. Multiple linear regression analysis was performed to determine the components influencing psychological resilience. The original regression model included all variables related to the outcome of the univariate analysis that were significant at the *P*-value of < 0.2 level: gender, ethnicity, sub-ethnicity, villages, number of siblings, religion, home ownership, quality of life, self-esteem and family environment. In the final model, psychological resilience was significantly associated with self-esteem, relationship and family dynamics, village location, number of siblings and ownership of residential property. According to the findings, one unit increase in self-esteem will increase the psychological resilience by 0.61 units (95% CI: 0.17, 1.06). In addition, one unit of relationships and family dynamics will increase the psychological resilience by 1.39 units (95% CI: 0.31, 2.47).

In contrast, participants residing in KOA Batu 12 had a psychological resilience score that was 14 units lower (95% CI: 4.69, 23.86) compared to those from KOA Bukit Lagong. Similarly, participants from KOA Ulu Batu had a psychological resilience score of 18 units lower (95% CI: 7.56, 28.02) than those from KOA Bukit Lagong. The participants who had fewer than four siblings showed psychological resilience scores that were 7 units lower (95% CI: 1.88, 11.11) compared to those who had four or more siblings. A significant relationship was also found between psychological resilience and owning residential property. Participants who owned their residential properties had a psychological resilience score of 8 (95% CI: 0.92, 14.16) units lower than those who did not own residences. The five significant variables described a 57% variation in psychological resilience score in the study sample (R^2^ = 0.57), while the remaining 43% was described by other factors that were not considered in this study.

## Discussion

Psychological resilience was helpful during the recent COVID-19 outbreak as it emphasised strengths rather than weaknesses (33). This study discovered that psychological resilience was associated with various traits, including where a person lived, the number of their siblings, whether they had been property owners, their self-esteem levels and their relationship with their families.

### Level of Psychological Resilience

The findings showed that during the COVID-19 pandemic, the mean psychological resilience of OA teens was 69.28 (SD = 14.52). The psychological resilience score was slightly lower than the survey conducted among Native Americans with mean score of 75.02 (SD = 13.43) during the same period (34). Our findings were also less favourable than prior COVID-19 research among Native Americans that involved educators and the elderly, with mean psychological resilience ratings of 78.3 (SD = 15.4) and 83.0 (SD = 15.4), respectively (35, 36). However, the outcomes were consistent with a global general population survey which found that over 25% of persons lacked psychological resilience, with younger people having lower psychological resilience (37). Similar findings were found in another study on the general population of Thailand, where more than 80% of participants had psychological resilience scores ranging from low to medium during the COVID-19 epidemic (38).

Resilience is multi-dimensional; many factors could affect the psychological resilience score, including living conditions and lockdown periods. Closeness and intimacy to living conditions improve psychological resilience as they find meaning. Connecting to a place related to better health outcomes in countries such as Canada and Australia gives people the strength to adjust to changes (39–41). However, in this study, the extended restriction period experienced by the suburban OA youth population may have contributed to a low psychological resilience similar to the general population during the COVID-19 pandemic, given that they have experienced environmental changes due to urbanisation. Duration of restriction can contribute to post-traumatic stress, and longer restriction period leads to emotional harm (35, 42).

### Factors Associated with Psychological Resilience

The location of villages, number of siblings, residential property ownership, level of self-esteem, and relationship and family dynamics are the major factors significantly associated with psychological resilience among OA during COVID-19. Compared to the KOA Bukit Lagong, residents of KOA Batu 12 and KOA Ulu Batu exhibited lower levels of psychological resilience. It could be due to the remote area settlements in KOA Bukit Lagong and getting tucked away in the forest’s depths, which designed a more conducive natural resilient habitat. These findings matched the observational research findings at KOA Kampung Tanjung Rambai in Selangor, Malaysia (43). Because their livelihoods depended on the ecology of the forest, the study found that the community maintained a link with their land, encouraging resource restraint with minimal disruptions and enhanced resilience. (43).

Resilience was lower in individuals with fewer than four siblings than those with more than four. More siblings equal more family members, and close family ties might boost psychological toughness. A study conducted on teenagers receiving institutional care in Northern and Central Portugal found that sibling ties can give security by helping people to feel accepted and understood through symmetry, resemblance and sharing of experiences (44). Sethi (46) states that since siblings play an integral role in our lives, having a sibling in college significantly raises the likelihood of the student developing psychological resilience. As they often grow up in the same family, their growth, happiness and resilience are connected as they spend most of their time together.

Another factor connected with psychological resilience was residential ownership. The strength of residential ownership was significantly lower than that of those who owned other properties. This is likely because youths living in extended families have a more robust problem-solving support system. Those who share a home but not own one have greater psychological resilience. As per the previous literature, the COVID-19 pandemic led to anxiety, tension and depression, but healthy relationships, communication and social support helped families cope with the crisis (46). International studies on family resilience have documented strong emotional relationships, efficient communication, coping skills and belief systems, especially spiritual or religious ones, which aid families in overcoming adversity. Moreover, multigenerational co-residence reduces adolescent parental stress, although non-resident fathers and other father figures have an impact on a child’s development in single-mother households (47).

This study also found an association between self-esteem and psychological resilience among OA youths. The OA community youths with high esteem were more optimistic, ambitious and yearned for success, which helped them overcome challenges and build psychological resilience. This finding is consistent with a study on psychological resilience among Australian aboriginal youths, which determined high self-esteem connected to positive psychosocial function across a spectrum of familial risk (48). Another study with a sizable sample of indigenous children in Western Australia found a positive correlation between psychological resilience and self-esteem. Community members expressed a wish for more initiatives to empower and educate at-risk indigenous children by identifying risks in their families and offering resources and support (49).

Relationship and family dynamics were also linked to OA’s psychological resilience during the pandemic. Although the potential negative impact of the pandemic on family dynamics and children is concerning, it is widely acknowledged that indigenous communities prioritise familial bonds, which could serve as a protective factor for young individuals in mitigating the adverse implications of the COVID-19 crisis (50). A study among indigenous Australians highlights the importance of family communication during a pandemic and illuminates Maori cultural values. Family members advise close and extended family through everyday digital activities (51).

This study highlighted that indigenous youths need strong relationships with land and nature, which may serve as a hidden strength or source of psychological resilience and well-being in urban contexts. To provide urban indigenous adolescents with opportunities to engage with nature and discover meaning, public health and local government should preserve, enhance and establish culturally significant natural areas (37). Psychological resilience is also associated with supportive relationships among family members and peers, and it can be learned or fostered through positive interactions. Programmes that identify and serve at-risk children at educational institutions or out-of-home care are potentially helpful for resilience building (52). The findings also indicated that fostering self-worth, arming children with the knowledge to make wiser decisions, and fostering pride in and familiarity with indigenous culture should be the main goals of resilience techniques.

### Strengths and Limitations

The strengths identified in this study are indigenous Malaysians’ psychological resilience determination during the COVID-19 pandemic and those factors overlooked by previous studies. Additionally, the initial level of psychological resilience among youths was confirmed, and particular aspects that could be prioritised to augment their resilience were identified. Third, this survey was constructed and pre-tested using a group of young people using a validated psychological resilience questionnaire with strong internal consistency and an alpha coefficient of 0.90.

Despite the strengths, there were also limitations. While certain OA communities have poor internet access, the digital research methodologies perform better in neighbourhoods with reliable internet service. Additionally, a comprehensive list of respondents’ phone numbers was absent from the survey. Selection bias resulted from the survey’s online and field execution (53). A second limitation includes coverage of only suburban OA villages in one Malaysian district, whereby the psychological resilience levels may differ between OA villages at different locations, especially in rural areas. So, future studies should include OA villages in rural and urban areas, particularly teenagers and young adults, to understand the resilience of Malaysian indigenous communities better. Next, this study’s results were only about the OA in Malaysia, excluding other indigenous people who may have their own culture and beliefs. Given that the study was conducted 2 years after the COVID-19 pandemic and throughout 9 months, the participants’ psychological resilience may have been impacted by the severity of the pandemic, its public health interventions and the evolving social distancing policies.

## Conclusion

In conclusion, the psychological resilience of OA is predicted by the village location, number of siblings, property ownership, self-esteem, relationship and family dynamics. An interdisciplinary approach considering the environmental and sociocultural elements is needed to develop psychological resilience solutions for a group. Enhancing resilience across multiple levels is needed (54). OA youths can be aided to become more resilient by improving their villages in nature, boosting their self-esteem, teaching them survival skills and strengthening their family ties. It can be done through culturally relevant approaches, avoiding a one-size-fits-all approach and imposing a Western biological programming framework on indigenous cultures. In this study, future studies are recommended to identify effective intervention programmes tailored to youths with low psychological resilience in the OA community and how psychological resilience changes over time, as it is irresponsible to ignore structural issues and the need for culturally appropriate resources while thinking crisis response principles apply everywhere.

## Figures and Tables

**Table 1:** Socio-demographic characteristics of the study participants (*N* = 158)

Variable	Mean (SD)	Frequency (%)
Age	20.96 (1.92)	
Gender		
Male		54 (34.2)
Female		104 (65.8)
Ethnic group		
Senoi		43 (27.2)
Proto-Malay		114 (72.2)
Negrito		1 (0.60)
Sub-ethnic		
Semai		29 (18.4)
Mah Meri		3 (1.9)
Temiar		11 (7.0)
Semang		1 (0.6)
Jakun		1 (0.6)
Semelai		4 (2.5)
Temuan		109 (69.0)
Villages		
Batu 12		68 (43.0)
Batu 16, Sungai Relang and Kemensah		3 (1.9)
Bukit Lagong		8 (5.1)
Kuang		44(27.8)
Sungai Buloh		21 (13.3)
Ulu Batu		14 (8.9)
Educational status		
Primary school		37 (23.4)
Secondary school		100 (63.3)
STPM/Pre-university		6 (3.8)
Certificate		2 (1.3)
Diploma		8 (5.1)
Degree/Master/PhD		1 (0.6)
No education/non-formal education		4 (2.5)
Marital status		
Single		114 (72.2)
Married		44 ( 27.8)
Divorced/widowed		0 (0.0)
Household income (monthly)		
< RM4,500		154 (97.5)
RM4,500–RM10,000		3 (1.9)
> RM10,000		1 (0.6)
Job-status		
Self-employed		12 (7.6)
Part-timer		12 (7.6)
Full-time		50 (31.6)
Not working		84 (53.2)
Family structure		
Live with both parents		124 (78.5)
Live with single parent		11 (7.0)
Live with others		23 (14.6)
Number of siblings		
Less than 4		36 (22.8)
Equal or more than 4		122 (77.2)
Religion		
Islam		87 (55.1)
Christian		9 (5.7)
Others		62 (39.2)
Ownership type of residential property		
House owner		133 (84.2)
Rental		11 (7.0)
Others		14 (8.9)

**Table 2:** Levels of psychological resilience, QOL, self-esteem, DASS and family environment of participants (*N* = 158)

Variable	Mean (SD)	Frequency (%)
Psychological resilience	69.28 (14.52)	
QOL	3.95 (0.76)	
General health	4.15 (0.69)	
Physical health	59.10 (10.43)	
Psychological	60.76 (16.83)	
Social relationship	71.54 (13.72)	
Environment	68.39 (13.29)	
Self-esteem	35.77 (4.94)	
Depression		
Normal		123 (77.8)
Mild to extremely severe		35 (22.2)
Anxiety		
Normal		121 (76.6)
Mild to extremely severe		37 (23.4)
Stress		
Normal		137 (86.7)
Mild to extremely severe		21 (13.3)
Family environment		
Togetherness and harmony	18.13 (3.10)	
Expression	16.01 (3.75)	
Relationship and family dynamic	18.83 (2.89)	
Conflict	16.55 (3.84)	
Religiosity and traditional practice	17.27 (3.18)	

**Table 3:** Factors associated with psychological resilience among the participants (*N*=158)

Variables	SLR^a^		MLR^b^		
	*b*^c^ (95%CI)	*P*-value	Adj. b^d^ (95%CI)	*t*-stat	*P*-value
Villages (KOA)					
Bukit Lagong	–	Ref.	–	–	Ref.
Batu 12	−15.81 (−26.20, −5.42)	0.003*	−14.27 (−23.86, −4.69)	−2.95	0.004*
Batu 16, Sungai Relang and Kemensah	−20.88 (−39.77, −1.99)	0.031*	−9.86 (−25.13, 5.42)	−1.28	0.204
Kuang	−16.21 (−26.92, −5.50)	0.003*	−8.43 (−17.53, 0.66)	−1.84	0.069
Sungai Buloh	−13.30 (−25.67, −0.94)	0.035*	−9.55 (−20.18, 1.09)	−1.78	0.078
Ulu Batu	−22.14 (−34.36, −9.93)	< 0.001*	−17.79 (−28.02, −7.56)	−3.44	< 0.001*
Number of siblings					
≥ 4	–	Ref.	–	–	Ref.
< 4	−6.76 (−12.11, −1.41)	0.014*	−6.50 (−11.11, −1.88)	−2.79	0.006*
Ownership type of residential property					
Others	–	Ref.	–	–	Ref.
House owner	−7.57 (15.59, 0.45)	0.064	−7.54 (−14.16, −0.92)	−2.26	0.026*
Rental	−8.12 (−19.62, 3.37)	0.165	−6.61 (−15.78, 2.56)	−1.43	0.156
Self-esteem	0.81 (0.36, 1.26)	< 0.001*	0.61 (0.17, 1.06)	2.74	0.007*
Relationship and family dynamic	2.13 (1.41, 2.85)	< 0.001*	1.39 (0.31, 2.47)	2.55	0.012*

Notes:

a Simple linear regression;

b Multiple linear regression (R^2^ = 56.7; The model reasonably fits well; Model assumptions are met; There is no interaction between independent variables and no multicollinearity problem);

c Crude regression coefficient;

d Adjusted regression coefficient;

* Statistical significance at α = 0.05
